# Effect of maternal hypothyroidism during pregnancy on insulin resistance, lipid accumulation, and mitochondrial dysfunction in skeletal muscle of fetal rats

**DOI:** 10.1042/BSR20171731

**Published:** 2018-07-03

**Authors:** Tongjia Xia, Xue Zhang, Youmin Wang, Datong Deng

**Affiliations:** 1 Department of Endocrinology, The First Affiliated Hospital of Anhui Medical University, No. 218 Jixi Road, Hefei, Anhui 230022, China; 2 Department of Endocriology, Anhui Medical University, Hefei, Anhui 230032, China

**Keywords:** fetal rats, hypothyroidism, insulin resistance, lipid accumulation, mitochondrial dysfunction

## Abstract

The present study aimed to investigate the effect of maternal hypothyroidism during pregnancy on thyroid function of the fetal rat. Female Sprague–Dawley rats were randomized into two groups. Propylthiouracil (PTU) group received PTU in drinking water for 6 weeks (*n*=90), normal group received normal drinking water (*n*=50). The pregnant rats were obtained and had a cesarean-section to get at gestational ages of 8.5, 13, and 21 days, following blood samples and skeletal muscle were obtained from fetal rats. Levels of thyroid hormone, insulin, mitochondrial protein, and adipokines were detected using ELISA. Western blotting was performed to analyze mitochondria and insulin signal transduction-related protein in fetal rat skeletal muscle. Immunostaining of Periodic Acid-Schiff (PAS) and Oil Red O was used to observe the accumulation of muscle glycogen and lipid in the fetal rat. The results showed that the levels of thyroid hormone, insulin, insulin signal transduction-related protein, mitochondrial, and adipokines increased with the fetus developed, but had no statistical differences in the PTU group compared with the normal group. In conclusion, pregnant rats with hypothyroidism had no influence on insulin resistance (IR), lipid accumulation, and mitochondrial dysfunction in skeletal muscle of the fetal rats.

## Introduction

Hypothyroidism is the most common thyroid disease, which is characterized by disordered circulating levels of thyroxine (T4), tri-iodothyronine (T3), and thyroid-stimulating hormone (TSH). Thyroid hormones are involved in regulating various physiological functions and pathological processes in the body, including changes in metabolism of carbohydrate, protein, and lipids, influence cell proliferation and development, and modulate response to other hormones [1–3]. Thyroid hormone is a key determinant of glucose homeostasis by regulating the balance of insulin [4], but hypothyroidism can disrupt hormonal balances and alter glucose and lipid metabolism to lead to insulin resistance (IR) [1,5,6]. It is also well established that hypothyroidism is a risk factor for IR. It has been reported that lipid deposition within non-adipose tissue, such as skeletal muscle, contributes to the development of IR [7]. In addition, previous studies have demonstrated that adipokines and thyroid hormones have some physiological effects in common including regulating energy expenditure and metabolism of glucose and lipids, which reflect an interplay involving both the thyroid axis and action of adipose tissue [8,9].

Thyroid dysfunction may affect the action of adipose tissue, and induce other metabolic dysregulation, such as IR [10], which indicates that mitochondria are the key players in the IR development. It is worth noting that mitochondrial dysfunction and/or reduced mitochondrial content can cause fatty acid oxidation in mitochondria, which can lead to the accumulation of fatty acyl-CoA and diacylglycerol. This interferes with the insulin signaling [11]. In skeletal muscle, enhancement of mitochondrial oxidant production promotes the development of IR, resulting in decreased insulin signaling and glucose transport via different pathways [2].

However, it is not clear whether pregnant rats with thyroid dysfunction could induce fetal thyroid dysfunction. Hence the present study was undertaken to explore the effect of pregnant rats with hypothyroidism on IR, lipid accumulation, and mitochondrial dysfunction in skeletal muscle of fetal rats. In our study, a stable rat model of hypothyroidism was first established by the treatment of propylthiouracil (PTU). We next evaluated serum levels of thyroid hormone in fetal rats and levels of maldondialdehyde (MDA), glutathione peroxidase (GSH-Px), superoxide dismutase (SOD), catalase (CAT), adiponectin, leptin, visfatin, resistin, Irisin and, TNF-α in gastrocnemius muscle homogenates, as well as proteins involved in mitochondrial oxidation and insulin signal transduction were detected. Furthermore, in fetal rat skeletal muscle, contents of muscle glycogen and lipid deposition were observed.

## Materials and methods

### Animals experiment

In the experiment, all Sprague–Dawley (4-week-old weighing 165 ± 5 g, males and females) were obtained from Experimental Animal Center of Anhui Medical University (Anhui, China). All experimental protocols were approved by the Institutional Animal Care and Use Committee of The First Affiliated Hospital of Anhui Medical University. The female rats were randomly divided into two groups. Hypothyroidism was induced in female rats (*n*=90) by the maintenance of 0.05% PTU in drinking water for 6 weeks (PTU group); control rats (*n*=50) receive an equivalent amount of water (normal group). After 6 weeks, rats in each group had free access to water. Sampling blood was taken from the orbital venous plexus of rats (*n*=6 each group) randomly selected from PTU group and normal group. Thyroid hormone was analyzed to validate the feasibility of the model. Then female rats with hypothyroidism and control group were kept in separate cages with mature male rats, and the day when a pessary was found was taken as day 0 of gestation (E0). In addition, pregnancy and spontaneous abortion rates were observed. After the mothers were fasted for 12 h, the embryo was taken out at gestational age of 8.5, 13, and 21 days (E8.5, E13, and E21) using a cesarean section. After a 12-h fast, blood sample was taken from fetal rats (as the above gestational age) to isolate serum and gastrocnemius muscle samples were collected snap-frozen in liquid nitrogen and stored at −80°C or fixed in 4% paraformaldehyde for follow-up study.

### Homeostatic model assessment

Homeostatic model assessment of IR (HOMA-IR) was used for assessment of IR by Matthews et al. [12] method. HOMA-IR is the product of the rat’s fasting insulin level times fasting glucose levels in mmol/l divided by 22.5 (HOMA = Insulin (μU/ml) × Glucose (mmol/l)/22.5).

### Fasting blood glucose, total cholesterol, triglycerides, and free fatty acids examination

Serum levels of fasting blood glucose (FBG), total cholesterol (TC), triglycerides (TG), and free fatty acids (FFA) were evaluated using an automatic biochemical meter (Beckman CX7; Beckman Coulter, CA, U.S.A.).

### ELISA analysis

Blood samples from female and fetal rats were collected for serum hormonal analyses. The serum levels of total T3, T4, TSH, and insulin were measured by using ELISA kits (Monobind, Inc., Costa Mesa, U.S.A.). The mitochondrial proteins and adipokines in gastrocnemius muscle homogenates (at gestational age of 8.5, 13, and 21 days) were evaluated using rat-specific commercially available ELISA kits according to the manufacturer’s instructions. The kits were purchased from the following companies respectively: SOD, GSH-Px, MDA, and CAT (Nanjing Jiancheng Institute of Biotechnology, Nanjing, China); adiponectin (Bionewtrans Pharmaceutical Biotechnology Co., Ltd, U.S.A.); leptin, TNF-α, resistin, visfatin (USCN Life Sciences Inc., Wuhan, China); Irisin (BioVision, Inc., Mountain View, CA).

### Western blot

Total protein (20 μg) from gastrocnemius skeletal muscle of PTU group and normal group was used for Western blot. Then equal protein samples were separated by SDS/PAGE (12% gel), transferred on to PVDF membranes, and blocked with 5% skim milk powder. Then, the membranes were incubated with primary antibodies as follows: rabbit polyclonal anti-peroxisome proliferator-activated receptor-γ co-activator-1α (PGC-1α, 1:2000, Abcam), rabbit anti-carnitine palmitoyltransferase 1 (CPT1, 1:1000), anti-glucose transporter 4 (GLUT4, 1:2000, Santa Cruz Biotechnology, CA, U.S.A.), rabbit anti-insulin receptor (IRC) substrate-1 (IRS-1, 1:1000, Upstate Biotechnology, Lake Placid, N.Y.), rabbit anti-IRC (1:1000, Abcam), rabbit anti-phosphoinositide 3-kinase (PI3K, 1:1000; Cell Signaling Technology, U.S.A.), β-actin (1:2000, Abcam, U.S.A.) were added at room temperature for 1 h. The results were quantitated with the Enhanced Chemiluminescence-Plus Western Blotting Detection System (Amersham, Cambridge, U.K.). The optical density of each band was normalized by β-actin optical density.

### Immunohistochemical staining

Immunohistochemistry was performed on the muscle samples from fetal rats. Tissue sections (5-µm-thick) were dewaxed, rehydrated, and repaired, then endogenous peroxidase activity was quenched using 3% hydrogen peroxide in methanol for 10 min. The sections were stained with Periodic acid-Schiff (PAS) (Sigma, Aldrich) or Oil Red O (Nova Ultra Oil Red O Stain Kit, IHC World, Woodstock, MD) according to manufacturers’ guidelines. Histology pictures were captured using a microscope with a digital camera (Nikon, Japan).

### Statistical analysis

All data were expressed as mean ± S.D. Statistical analyses were performed using the SPSS 19 statistical software program. Statistical significance was evaluated with an unpaired two-tailed Student’s *t*test or by one-way ANOVA, followed by Dunnett’s test. *P*<0.05 was considered statistically significant.

## Results

### Clinical characteristics of female rats

Our first experiments were designed to determine if the hypothyroidism animal models were successful. Thyroid hormone levels of female rats (PTU groups, *n*=6; normal group, *n*=6) are shown in Table 1, levels of serum T3 and T4 were lower in PTU group compared with the normal group (*P*<0.05). Levels of TSH were significantly increased in PTU groups compared with the normal group (*P*<0.05). These results suggested an animal model of hypothyroidism was set up successfully. Examination of pregnancy rate of female rats was not markedly different between each group (PTU groups, *n*=78; normal group, *n*=44), whereas a significant increase in miscarriage rate of PTU group was observed (*P*<0.05 compared with the normal group, Table 2).

**Table 1:** The results of making hypothyroidism of female rats in different groups

Groups	T3 (ng/ml)	T4 (μg/l)	TSH (μIU/l)
Normal group (*n*=6)	124.75 ± 5.86	226.51 ± 7.90	1073.0 ± 75.32
PTU group (*n*=6)	66.53 ± 6.92*	173.67 ± 5.05*	1473.0 ± 83.44*

* *P*<0.05, compared with normal group.

**Table 2:** The results of pregnancy rate and miscarriage rate of female rats in different groups

Groups	Pregnancy rate (%)	Miscarriage rate (%)
Normal group (*n*=44)	48.7	0
PTU group (*n*=78)	46.9	17.8*

* *P*<0.05, compared with normal group.

**Figure 1:**
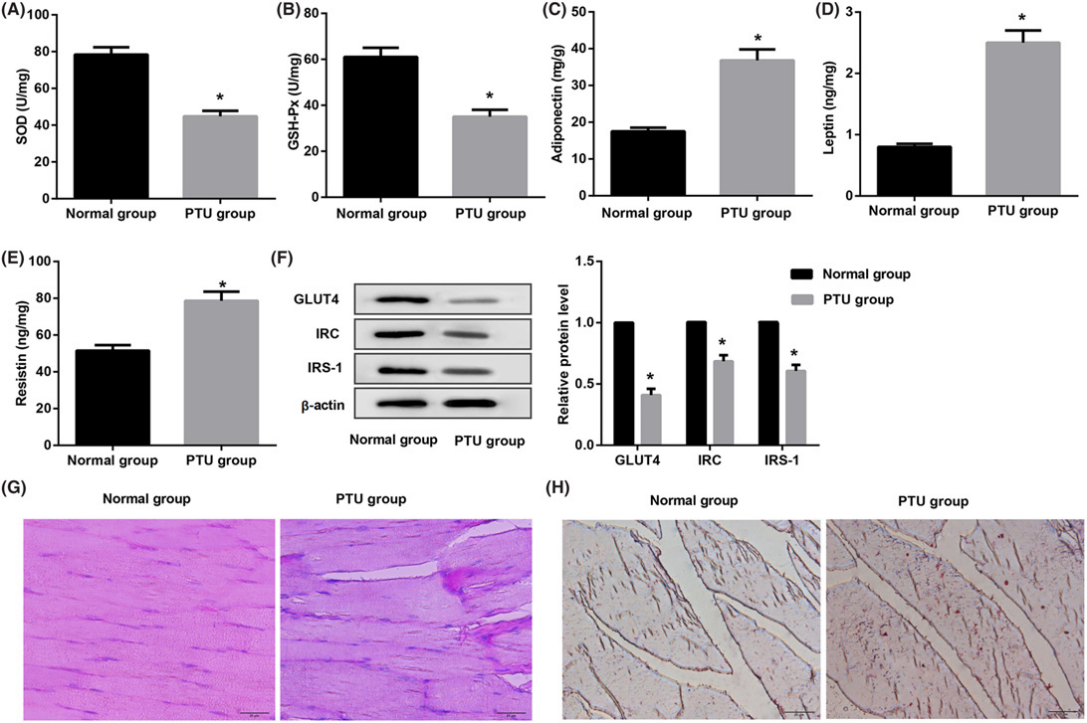
Effect of hypothyroidism on skeletal muscle in adult female rats ELISA analysis of (**A**) GSH-Px, (**B**) SOD, (**C**) adiponectin, (**D**) leptin, and (**E**) resistin in skeletal muscle homogenates from the PTU groups and the normal groups. (**F**) Western blot analysis of GLUT4, IRC, and IRS-1 proteins from skeletal muscle of adult rats. Representative images stained with PAS (**G**) showing glycogen accumulated in rat adult skeletal muscle (amaranth) and Oil Red O (**H**) showing lipid deposition in rat fetal skeletal muscle (Red). Scale bar = 20 μm.

In addition, PTU-induced hypothyroid adult female rats in alteration of IR, lipid accumulation, and mitochondrial dyfunction were assessed. As shown in Figure 1A,B, levels of SOD and GSH-Px in homogenate of skeletal muscle from adult female rats with hypothyroidism were significantly decreased than that in the normal groups (*P*<0.05). Meanwhile, we found that PTU rats had an obviously increased in adipokines-adiponectin (*P*<0.05, Figure 1C), leptin (*P*<0.05, Figure 1D), and resistin (*P*<0.05, Figure 1E) level compared with normal groups. Furthermore, expression levels of GLUT4, IRS-1, and IRC proteins were decreased in adult female rats with hypothyroidism than normal group (*P*<0.05, Figure 1F). Accordingly, the positive rate of PAS and Oil Red O stain in the adult rats from PTU group was increased compared as that in the normal group (Figure 1G,H). These data indicate that hypothyroidism promotes lipid accumulation and mitochondrial dysfunction of skeletal muscle in adult rats.

### Clinical characteristics of fetal rats

Our next experiments were intended to detect the effect of pregnant rats with hypothyroidism on fetal rats. At a specified point-in-time, fetal rats were obtained from pregnant rats of PTU and normal group. Then, thyroid function parameters of fetal rats are shown in Table 3, which demonstrates that thyroid hormone levels (T3 and T4) and TSH were increased with fetal growth. Level of T3 was lower in PTU group compared with normal group, whereas TSH was higher (embryo of 21 days, Table 3). T4 did not differ significantly between PTU and normal group (embryo of 21 days, Table 3). The FBG, fasting insulin (FINS), HOMA-IR, TG, and TC increased as the fetus developed in both the PTU group and the normal group (Table 4), and PTU group had increased TC in embryo of 21 days (*P*<0.05, Table 4).

**Table 3:** Thyroid function parameters of fetal rats at different times in different groups

	Groups	T3 (ng/ml)	T4 (μg/l)	TSH (μIU/l)
	E8.5 (*n*=6)	0	0	0
Normal	E13 (*n*=6)	0	0	0
	E21 (*n*=6)	0.003 ± 0.0008†	0.020 ± 0.0056†	56.230 ± 5.3200†
	E8.5 (*n*=6)	0	0	0
PTU	E13 (*n*=6)	0	0	0
	E21 (*n*=6)	0.001 ± 0.0004*,†	0.011 ± 0.0068†	72.480 ± 5.6520*,†

* *P*<0.05, compared with normal group at different times.

† *P*<0.05, compared with E8.5 in the same group.

**Table 4:** Serum biochemical parameters of fetal rats at different times in different groups

	Groups	FBG (mmol/l)	FINS (mU/l)	HOMA-IR	TG (mmol/l)	TC (mmol/l)
	E8.5 (*n*=6)	0.91 ± 0.10	3.87 ± 0.15	0.17 ± 0.05	0.11 ± 0.02	0.91 ± 0.15
Normal	E13 (*n*=6)	1.29 ± 0.32	4.54 ± 0.53	0.26 ± 0.04	0.14 ± 0.05	1.14 ± 0.33
	E21 (*n*=6)	2.01 ± 0.49†	6.29 ± 0.68†	0.56 ± 0.10†	0.21 ± 0.03†	1.31 ± 0.23†
	E8.5 (*n*=6)	0.94 ± 0.11	3.88 ± 0.46	0.19 ± 0.06	0.12 ± 0.03	0.91 ± 0.20
PTU	E13 (*n*=6)	1.47 ± 0.59	4.77 ± 0.69	0.31 ± 0.04†	0.16 ± 0.02	1.21 ± 0.27
	E21 (*n*=6)	2.34 ± 0.32†	7.07 ± 0.90†	0.73 ± 0.16†	0.29 ± 0.03†	1.54 ± 0.13*,^†^

* *P*<0.05, compared with Normal group at different times.

† *P*<0.05, compared with E8.5 in the same group.

### Levels of MDA, GSH-Px, SOD, and CAT in skeletal muscle

**Figure 2:**
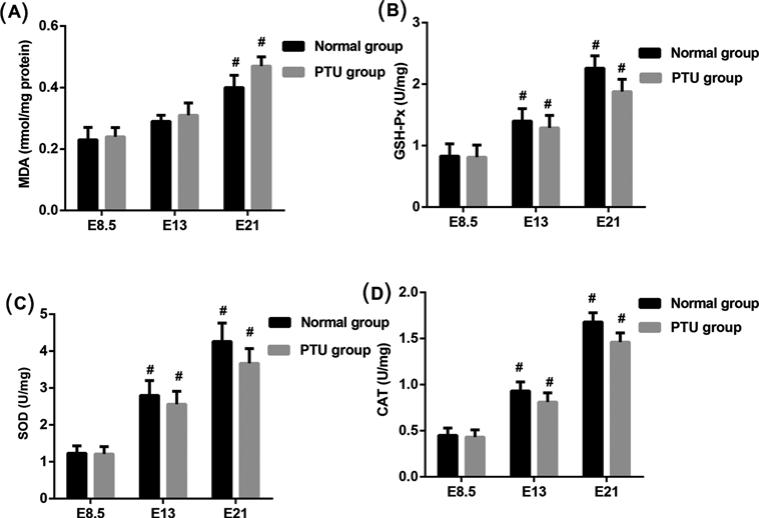
Assessment of adipokines in homogenate of skeletal muscle from fetal rats At gestational age of E8.5, E13, and E21, ELISA analysis of (**A**) MDA, (**B**) GSH-Px, (**C**) SOD, and (**D**) CAT in the PTU groups and the normal groups. ^#^*P*<0.05 compared with E8.5 groups.

The important components of the antioxidative enzymes play a key role in endogenous defense mechanism, such as SOD, CAT, GSH-Px [13]. To explore the effect of hypothyroid on mitochondrial function in skeletal muscle of fetal rats, ELISA was performed to detect the levels of MDA, GSH-Px, SOD, and CAT in homogenate of skeletal muscle from fetal rats (Figure 2). Levels of MDA were significantly increased in E21 group (*P*<0.05) but no statistical differences in E13 group compared with E8.5 group. Levels of GSH-Px, SOD, and CAT were higher in E13 and E21 group (*P*<0.05 compared with E8.5 group). In addition, there were no statistical differences in levels of MDA, GSH-Px, SOD, and CAT between the PTU groups and the normal groups in each gestational age.

### Serum levels of adipokines

**Figure 3:**
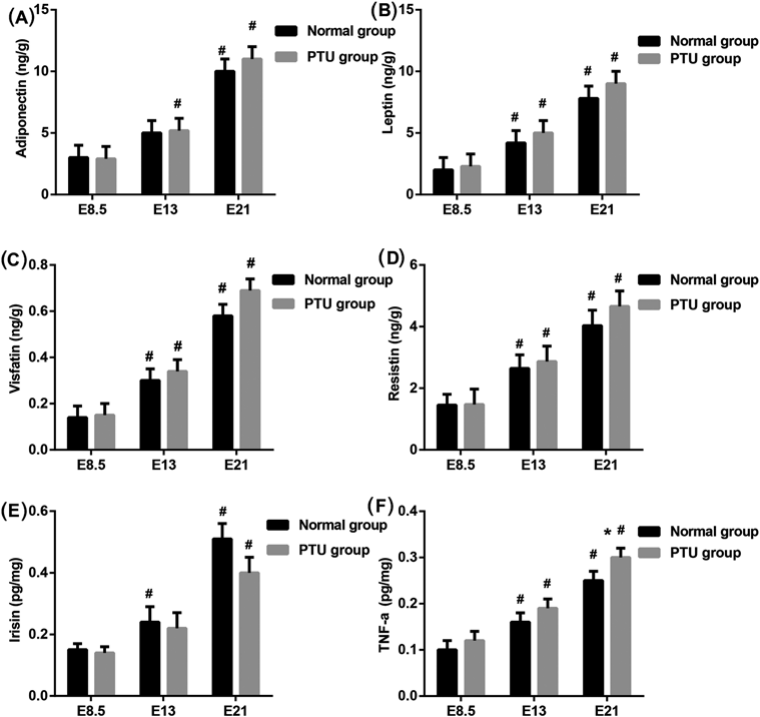
Detection of the adipokines in skeletal muscle of fetal rats ELISA analysis of (**A**) adiponectin, (**B**) leptin, (**C**) visfatin, (**D**) resistin, (**E**) irisin, and (**F**) TNF-α in homogenate of skeletal muscle of fetal rats from the PTU group and the normal group mothers. *P<0.05 compared with normal group at different time; ^#^*P*<0.05 compared with E8.5 in the same group.

It is reported that thyroid dysfunction effect on metabolism of adipokines [14]. We next examined the adipokines in skeletal muscle of fetal rats. Except adiponectin in E13 of normal group and irisin in E13 of PTU, E13 and E21 of PTU and normal group with significantly higher levels of adiponectin (Figure 3A), leptin (Figure 3B), visfatin (Figure 3C), resistin (Figure 3D), irisin (Figure 3E), and TNF-α (Figure 3F) than that in E8.5, separately (*P*<0.05). However, there was no significant difference in the levels of adipokines between the PTU and normal group.

### Effects of hypothyroidism on mitochondria and insulin signal transduction-related protein in rat fetal skeletal muscle

**Figure 4:**
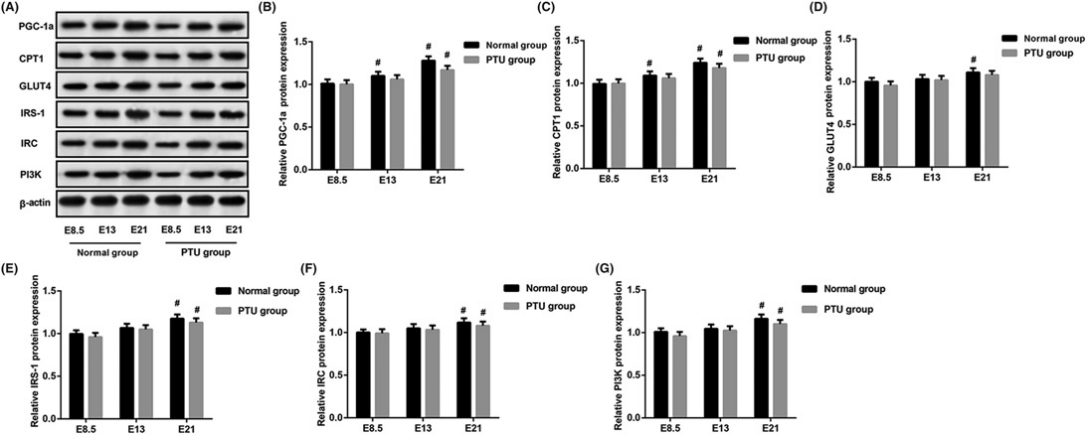
Assessment of mitochondria and insulin signal transduction-related protein in skeletal muscle of fetal rats (**A**) Western blot analysis of PGC-1α, CPT1, GLUT4, IRS-1, IRC, and PI3K proteins from fetal skeletal muscle. (**B–G**) The gray intensity of the above protein. ^#^*P*<0.05 compared with E8.5 in the same group.

It is reported that PGC-1α regulates lipogenesis, lipid accumulation, and substrate oxidation in skeletal muscle [15]. CPT deficiency is common disorder of mitochondrial fatty acid oxidation. To further characterize the effects of hypothyroidism on mitochondria and IR, we tested proteins involved in mitochondrial oxidation and insulin signal transduction by Western blot analysis (Figure 4A). The results showed that mitochondria-related proteins (PGC-1α and CPT1) were remarkably increased in E21 of PTU group and the normal group compared with their E8.5 (*P*<0.05, Figure 4B,C), but no statistical differences between the PTU group and the normal group in each gestational age. Furthermore, expression levels of GLUT4, IRS-1, IRC, and PI3K proteins were increased in E21 than in E8.5 in two groups (except GLUT4 in PTU group) (*P*<0.05, Figure 4D–G). However, the PTU group and the normal group had no difference.

### Muscle glycogen and lipid deposition in skeletal muscle of fetal rats

**Figure 5:**
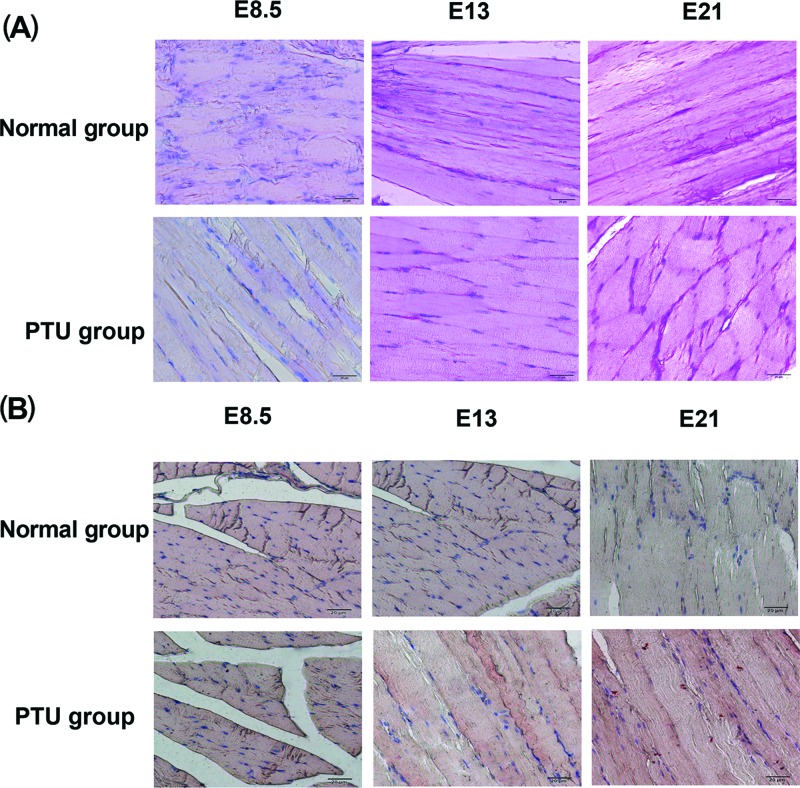
Analysis of muscle glycogen and lipid deposition in skeletal muscle of fetal rats (**A**) Representative images stained with PAS showing glycogen accumulated in rat fetal skeletal muscle (amaranth). Scale bar = 20 μm. (**B**) Representative images stained with Oil Red O showing lipid deposition in rat fetal skeletal muscle (red). Scale bar = 20 μm.

To analyze the contents of muscle glycogen and lipid deposition, immunohistochemistry stain of PAS and Oil Red O was performed on the muscle samples of fetal rats. PAS stain showed that the PTU groups and the normal groups had glucogen within the cytoplasm at E8.5, E13, and E21, and the positive rate of PAS stain was increased as the growth of fetuses (Figure 5A). Besides, tissues were stained with Oil Red O. As shown in Figure 5B, E21 of PTU groups had a small excess of lipid accumulation. But in contents of muscle glycogen and lipid deposition, no obvious difference between the PTU groups and the normal groups was observed at the same gestational age.

## Discussion

Thyroid dysfunction results in alterations of glucose and lipid metabolism which causes a series of pathophysiological changes. It is reported that mothers of congenitally hypothyroid infants had a lower T3 resin uptake (T3RU) concentrations [16]. In our study, we tried to observe the thyroid function in fetal rats from pregnant rats with hypothyroid. In the present study, the female rats were treated with PTU showed a significant decrease in serum levels of T3 and T4 but increase TSH, which demonstrated a successful modeling of hypothyroidism. Furthermore, PTU-induced hypothyroid adult female rats in SOD and GSH-Px level was significantly decreased, but adipokines-adiponectin, leptin, and resistin was elevated, expression levels of GLUT4, IRS-1, and IRC proteins were decreased. Meanwhile the positive rate of PAS and Oil Red O stain was increased than that in the normal groups. These data indicate that hypothyroidism promote lipid accumulation and mitochondrial dysfunction of skeletal muscle in adult rats.

An increasing number of researches suggest that maternal hypothyroidism could increase the risk of fetal thyroid dysfunction [17–19]. It has been reported that chronic alterations in thyroid status especially cause oxidative damage and lipid peroxidation in skeletal muscle of mice [20]. Additionally, the results from rat demonstrated that hyperthyroidism increased lipid metabolism in skeletal muscle [21]. On the basis of successful modeling, pregnant rats were obtained and a relatively high miscarriage rate showed in rats with hypothyroidism. Data from pregnant rat model indicate that hypothyroidism significantly increase the risk of miscarriage. Furthermore, at embryo of 21 days, T3 and TSH showed a significant change in PTU group compared with normal group, and levels of T3, T4, and TSH were increased with fetal growth. These data seem to indicate that the pregnant rats with hypothyroidism can increase the likelihood of abnormal thyroid function in fetal rats.

Various studies reveal that patients with hypothyroidism have an increased level of the hormone insulin [22–24]. In addition, abnormal levels of thyroid hormones and TSH in patients are likely to present with high incidences of IR, type 2 diabetes, and cardiovascular disorders [25,26]. We observed an observable rise in FBG, FINS, and HOMA-IR as the fetus in PTU group and the normal group, but not differ significantly between the two groups. This might be due to the effect of IR in fetal rats depending on maternal gestational age. IR is a disorderly state of glucose homeostasis in which insulin produced cannot meet the expected biological effects in the liver, muscle, adipose tissue, and other body tissues [5,7]. In hypothyroidism, peripheral IR cause in skeletal muscle and adipose tissue has been suggested, and skeletal muscle plays a key role accounting for most of the whole body glucose use [27]. In our study, we found that the levels of IRS-1, IRC, and PI3K proteins from rat fetal skeletal muscle were increased in E21 of the PTU group and the normal group but that no difference between two groups. Furthermore, in contents of muscle glycogen, no obvious difference between the PTU groups and the normal groups was observed at the same gestational age. These results indicate that maternal hypothyroidism have no effect on IR of fetal rats.

The abnormal composition and the transport of lipoproteins have been reported in thyroid diseases [14,28]. T4 therapy can considerably improve the lipid profile when in a TSH-suppressive dose and there has been a relationship between the changes in lipoproteins and changes in free T4 levels [28].

The research indicates that TH could alter muscle performance and heat production, and thus affect whole-body glucose homeostasis [29]. The role of adipocytokines in cross-communication between adipose and muscle tissue has been proposed [30]. In our study, levels of TG and TC were significantly higher in E21 of the PTU group and the normal group when compared with that in E8.5 and PTU group had increased TC in embryo of 21 days. As well as E21 of PTU and normal group had significantly higher levels of adiponectin, leptin, visfatin, resistin, irisin, and TNF-α than that in E8.5 but there was no markedly difference in levels of adipokines between PTU and normal group at any of the above gestational age. Beyond that, the positive rate of PAS stain was no obvious difference between the PTU groups and the normal groups. Above research result indicates that the condition of lipid accumulation in skeletal muscle of fetal rats is not affected by maternal hypothyroidism during pregnancy.

Thyroid hormones have been demonstrated to be a major regulator of mitochondrial bioactivity [31,32]. It is reported that thyroid hormones can induce alteration of mitochondrial membrane potentials, which present the functional status of mitochondria [33,34]. In our present study, there were no statistical differences in levels of mitochondria-related proteins between the PTU groups and the normal groups in each gestational age. These data reveal that maternal hypothyroidism during pregnancy will not cause mitochondrial dysfunction in fetal rats.

In conclusion, we have described an increased insulin and lipids levels, as well as enhancive mitochondrial function as growth of the fetal. But all the data suggest that maternal hypothyroidism during pregnancy is not influence upon IR, lipid accumulation, and mitochondrial dysfunction in skeletal muscle of fetal rats.

## Abbreviations

CAT: catalase
CPT1: carnitine palmitoyltransferase 1
FBG: fasting blood glucose
GLUT4: glucose transporter 4
GSH-Px: glutathione peroxidase
HOMA-IR: homeostatic model assessment of insulin resistance
IR: insulin resistance
IRC: insulin receptor
IRS-1: insulin receptor substrate-1
MDA: maldondialdehyde
PAS: periodic acid-Schiff
PGC-1α: peroxisome proliferator-activated receptor-γ co-activator-1α
PI3K: phosphoinositide 3-kinase
PTU: propylthiouracil
SOD: superoxide dismutase
TC: total cholesterol
TG: triglyceride
TSH: thyroid-stimulating hormone
T3: tri-iodothyronine
T4: thyroxine
